# Genotypic Identification of Trees Using DNA Barcodes and Microbiome Analysis of Rhizosphere Microbial Communities

**DOI:** 10.3390/genes15070865

**Published:** 2024-07-01

**Authors:** Liliana Hopkins, Kayla Yim, Ana Rumora, Melissa F. Baykus, Luisa Martinez, Luis Jimenez

**Affiliations:** Biology and Horticulture Department, Bergen Community College, 400 Paramus Road, Paramus, NJ 07652, USA; llhopkins145705@me.bergen.edu (L.H.); kyim142129@me.bergen.edu (K.Y.); arumora@me.bergen.edu (A.R.); mbaykus@me.bergen.edu (M.F.B.); lmartinez144129@me.bergen.edu (L.M.)

**Keywords:** Acidobacteriota, Ascomycota, Actinomycetota, Bacteroidota, Basidiomycetes, DNA barcodes, 16S rRNA, ITS, next-generation sequencing, Pseudomonadota, rhizosphere

## Abstract

DNA barcodes can provide accurate identification of plants. We used previously reported DNA primers targeting the internal transcribed spacer (ITS1) region of the nuclear ribosomal cistron, internal transcribed spacer (ITS2), and chloroplast *trnL* (UAA) intron to identify four trees at Bergen Community College. Two of the four trees were identified as *Acer rubrum* and *Fagus sylvatica*. However, *Quercus* was only identified at the genus level, and the fourth tree did not show similar identification between barcodes. Next-generation sequencing of 16S rRNA genes showed that the predominant bacterial communities in the rhizosphere mainly consisted of the Pseudomonadota, Actinomycetota, Bacteroidota, and Acidobacteriota. *A. rubrum* showed the most diverse bacterial community while *F. sylvatica* was less diverse. The genus *Rhodoplanes* showed the highest relative bacterial abundance in all trees. Fungal ITS sequence analysis demonstrated that the communities predominantly consisted of the Ascomycota and Basidiomycota. *Quercus* showed the highest fungi diversity while *F. sylvatica* showed the lowest. *Russula* showed the highest abundance of fungi genera. Average similarity values in the rhizosphere for fungi communities at the phylum level were higher than for bacteria. However, at the genus level, bacterial communities showed higher similarities than fungi. Similarity values decreased at lower taxonomical levels for both bacteria and fungi, indicating each tree has selected for specific bacterial and fungal communities. This study confirmed the distinctiveness of the microbial communities in the rhizosphere of each tree and their importance in sustaining and supporting viability and growth but also demonstrating the limitations of DNA barcoding with the primers used in this study to identify genus and species for some of the trees. The optimization of DNA barcoding will require additional DNA sequences to enhance the resolution and identification of trees at the study site.

## 1. Introduction

A comprehensive understanding of the ecosystem requires integrated and dimensional experimental research. Land plant systems must be analyzed within the context of their environment [1]. A bottom-up approach might include a study of both genetic composition and microbial community. Modern gene sequencing technologies and databases promote plant DNA barcoding as an efficient taxonomic and phylogenetic method [2,3,4]. This is extremely important due to the threat of extinction and the emerging environmental changes around the world. Furthermore, this is especially critical when plant and tree inventories are mostly performed based on morphological characteristics, such as leaf and fruit morphology, which can be misleading and might have underestimated the genetic diversity. The benefits of plants’ genotypic-based taxonomy include improved specificity and accuracy for identification, discrimination, and phylogenetic purposes [2,3,4,5,6,7,8,9,10]. It can also help to understand the distribution and evolution of plants in the environment regarding not only the interaction between plant species but also their genetic diversity and community structure. Factors such as climatic conditions, topography, soil nutrients, etc., are very important for determining plant community structure and diversity on a global and regional scale [1,3,5,6].

There are several potential genes available for land plant taxonomic classification. An ideal barcode system must be a DNA region variable enough to discriminate between species [6]. It should also have all the phylogenetic information to provide for the identification of the different taxonomic levels such as species, genus, family, order, class, and phylum. The sequence must also have conserved regions that can be amplified and sequenced. The size of the DNA region should not be too long so it can be amplified in a consistent, reliable, and accurate way. However, there are no sequences available that can fulfill all these requirements. Evidently, a single marker sequence cannot provide enough information for lower taxonomical level discrimination and identification [6].

The internal transcribed spacer (ITS1) region of the 18S–5.8S–26S nuclear ribosomal cistron is commonly used as a fungal and plant species barcode. It exhibits the potential to be used as a universal eukaryotic barcode. However, because of the possible amplification with fungi, DNA results can result in the wrong identification [7]. Another problem is that multiple and divergent copies can be present in the plant genome, complicating the sequencing and interpretation of the results. The ITS subregion, ITS2, may be used as a plant-specific barcode [8]. Despite the smaller size, ITS2 sequences provide highly specific interspecific divergence, leading to the identification at the genus and species levels. Furthermore, the levels of fungal co-amplification were significantly reduced [9]. Studies classified this gene as a supplementary confirmation sequence [9]. DNA metabarcoding (identification of plants in environmental samples) was reported using a combination of two primer pairs, ITS2F-ITSp4 and ITSp3-ITSu4. The taxonomic composition of the plant community varied between primer pairs, but using both sets of primers overcame the bias in the recovery of different taxonomic groups.

Different regions of the plastid genome have also been used to identify several plant species. For instance, *rbcLa*, *matK*, and *rbcL* genes are commonly used for genotypic testing [6]. This combination of barcodes was applied to 296 species of woody trees, shrubs, and palms found in the Forest Dynamics Plot on Barrio Colorado Island, Panama, with >98% correct species identification [3]. Using the results from the three different barcodes, they were able to develop a phylogenetic analysis of the different species identified to determine whether they were randomly distributed and closely or distantly related by chance. However, in some studies, this combination of barcodes did not provide proper identification and discrimination for different *Quercus* species in Italian tree flora due to the lack of sequence variation suitable for barcoding [5]. Nevertheless, accurate species identification from the genera *Acer*, *Pinus*, and *Populus* was reported.

A chloroplast *trnL* (UAA) intron can be also employed with the recommended ribosomal genes to maximize accuracy and reduce potential bias [4]. Primers have a high resolution in specific contexts, including common plant organisms [10]. Zeng et al. [4] demonstrated the use of this barcode to identify 11 tree species in Chinese subtropical forests. Furthermore, they successfully estimated the relative proportion of each species in mixed fine root samples. Because there is no barcode sequence that will identify all plants, nuclear ITS and chloroplast sequences, when used simultaneously, potentially create a streamlined and reliable DNA barcode procedure for land plant identification, which can overcome the deficiencies of phenotypic tests [6,8].

The biological processes of land plants must be studied in relation to their supporting microbial communities in the rhizosphere [11,12,13,14,15]. Land plants depend on their symbiotic relationships with bacteria and fungi in their respective rhizosphere microbiomes [1,11,12,13]. Depending on the plant species, different bacteria and fungi enable various essential metabolic reactions such as nitrogen fixation, phytohormone production, defense against plant pathogens, and the liberation of nutrients (phosphorus and sulfur) to sustain plant viability and growth [1,16,17,18,19]. Amplicon sequencing of 16S rRNA genes has characterized bacterial diversity in soils and rhizosphere at the individual, species, and community levels [19,20,21,22]. The bacterial diversity in the rhizosphere is reduced when compared to soils because plants and trees select members of the rhizosphere microbiome from soil communities based on their nutritional requirements [23]. However, the higher microbial biomass and activity in the rhizosphere is due to the high concentration of organic compounds released as plants exudates, which reduced soil pH values to be more acidic than in bulk soils [11,14,19]. Microbial community composition and structure in the rhizosphere can be determined by, among other factors, plant genotype, root exudates, pH, soil type, growth conditions, and agricultural practices [1,14,17,18,19]. Organic compounds enter the soil either by dead organic matter, e.g., leaf litter and dead roots, or root exudates. In temperate forests, carbon enters the soil mostly through root exudates [1]. The chemical composition of root exudates is very diverse and will be selected for specific bacteria based on the type of tree [1,11,14]. On the other hand, trees promote variable microbial communities to provide their nutrition, but the soil is the reservoir for the recruitment of specific bacterial taxa [1,11,24,25].

In addition to bacteria, ground vegetation and trees form symbiotic associations, such as ectomycorrhiza (EC), with fungi develop mycorrhiza [1,19,26,27,28,29,30]. The development of a network of mycelia extending the surface area of the plant roots provides a significant enhancement for the acquisition of nutritional requirements such as water, nitrogen, phosphates, minerals, etc. The rhizosphere contains a diverse fungi community consisting of saprotrophic and EC fungi. They exhibit a very different carbon metabolism with the EC fungi obtaining their carbon compounds from the host tree, while saprophytic fungi rely on the decomposition of organic matter.

An impediment that genetic analysis of environmental microbial communities face is a lack of information in genetic databases [31,32]. Large numbers of sequences have been reported not to match known bacteria and fungi phylum, class, order, family, genus, and species [7,15,19,20,21,24,31,32]. Below the class level, metagenomic analysis with amplicon sequencing is often uninformative. With the current available information, however, studies can determine the most abundant taxonomic levels in the rhizosphere. When studied in conjunction with an accurately identified and related plant, this information reveals potential functional relationships between plants, bacteria, and fungi [14,15,17,18,20].

There are approximately 85 trees at Bergen Community College (BCC). None of them has been genetically identified, nor have their rhizosphere microbial communities been characterized. Trees are important to sustain life and biodiversity in the environment [33]. Planting a tree on campus provided green spaces where students, faculty, and workers relax and interact with each other. They also reduce energy costs by creating shade covering, cleaner air (reducing carbon dioxide in the atmosphere), and cleaner water. Student involvement in the planting and maintenance of trees encourages the learning of a more sustainable way of life and the appreciation of the different contributions of plants and trees to a healthy and productive ecosystem.

Fungi communities in BCC soils were previously studied by cloning of ITS1 sequences detected in bulk soil DNA by a polymerase chain reaction (PCR) [7]. Most of the identified sequences were aligned to the phyla Ascomycota and Basidiomycota. However, at the genus level, only 15 clones were analyzed, with unidentified fungal sequences showing the highest frequencies (67%). The only fungal species identified were *Curvularia trifolii*, *Penicillium restrictum*, and *Phoma herbarum*. Soil samples from similar locations were also analyzed using next-generation sequencing of 16S rRNA genes to describe the bacterial community [24]. The results showed that the most abundant phyla were Actinomycetota (33.76%), Pseudomonadota (25.60%), Chloroflexota (9.70%), Acidobacteriota (8.98%), and Planctomycetota (6.10%). Dominant bacteria were mostly related to Actinomycetota families and genera and Acidobacteriota classes.

To understand the genetic diversity of the trees on campus and characterize the bacterial and fungi community in the rhizosphere, we developed a DNA barcoding protocol using previously reported primers targeting the ITS1, ITS2, and *trnL* (UAA) DNA sequences and analyzed the microbial communities using next-generation sequencing of ribosomal genes to start building a genetic map of trees and their bacterial and fungal communities to make inferences about the functional relationships that connect them to maximize plant and microbial growth.

## 2. Materials and Methods

### 2.1. Soil Sampling

Soil samples from the rhizosphere of four trees were aseptically collected as previously described from different locations at the BCC campus located in the city of Paramus, NJ, USA [7]. The selection was based on assumed phenotypic differences and locations. Samples were labeled as Trees (T) 1 to 4.

### 2.2. DNA Extraction and PCR Amplification from Rhizosphere Soils

Microbial DNA from rhizosphere soils was extracted using the ZR Soil Microbe DNA MiniPrep Protocol (Zymo Research, Irvine, CA, USA) as previously described [7]. Extractions were performed in duplicates. DNA concentration was determined using the Qubit^®^ dsDNA HS assay as previously described by Jimenez et al. [34]. To analyze the quality of the extracted DNA, PCR amplification was performed using primers 341f (CCTACGGGNGGCWGCAG) and 785r (GACTACHVGGGTATCTAATCC), which amplified the V3–V4 fragment of the 16S rRNA gene with a size of approximately 465 base pairs (bps) [35]. Reaction conditions were as follows: 95 °C for 5 min, followed by 25 cycles consisting of denaturation at 95 °C for 40 s, annealing at 55 °C for 2 min, and extension at 72 °C for 1 min. After the 25 cycles were completed, a final extension step at 72 °C for 7 min was added to the reaction. Fungal internal transcribed spacer regions (ITS) were analyzed for PCR, targeting a 640 bp fragment [36].

Ready-To-Go (RTG) PCR beads (GE Healthcare, Buckinghamshire, UK) were used for each PCR reaction volume as previously described [34]. Reaction mixtures were added to a T100TM thermal cycler (Bio-Rad Laboratories, Hercules, CA, USA) or Mastercycler thermal cycler (Eppendorf Scientific, Westbury, NY, USA). After PCR amplification, amplicon detection was analyzed by gel electrophoresis using the FlashGel system (Lonza Inc., Rockland, ME, USA) as described by Jimenez et al. [34]. A FlashGel DNA Marker (Lonza Inc., Rockland, ME, USA) with fragment sizes ranging from 100 bp to 4 kilobases (kbs) was used to determine the presence of the correct DNA fragments.

### 2.3. DNA Extraction from Leaf Samples

Leaf samples from each tree were aseptically cut into small pieces and grounded with mortar and pestle. A total of 100 to 200 microliters of sterile water were added to the leaves. A total of 0.05 to 0.2 g of the leaf paste was added to the BashingBead^TM^ Lysis Buffer (Zymo Research, Irvine, CA, USA). Samples were mixed for 10 min to ensure lysis. After mixing, centrifugation was performed at 10,000× *g* for 3 min. Plant DNA was extracted as described by Jimenez et al. [7].

### 2.4. PCR Amplification and DNA Barcoding for Plant DNA

To optimize accuracy, specificity, and reliability in our land–plant taxonomic classification method, we targeted one chloroplast and two ribosomal genes. The internal transcribed spacer (ITS) region of the 18S–5.8S–26S nuclear ribosomal cistron is a potential universal barcode for eukaryotic organisms [7]. To amplify the ITS sequence, the ITS1 and ITS4 primers target an estimated 640 bp fragment [36]. We used the reaction constituents and conditions previously described [7]. Its subregion, the ITS2, was used as a supplementary, plant-specific barcode [9,37]. The ITS2F and ITS3R primers were used for sequence amplification [37]. The ITS2 PCR reaction conditions used were previously described [37]. This study used the chloroplast *trnL* (UAA) intron gene as a supplementary, plant-specific barcode. We followed the reaction conditions previously detailed by Taberlet et al. [10], using the c and d primers to amplify a 456 bp fragment. DNA sequencing reactions of the ITS1, ITS2, and UAA DNA fragments were performed by Azenta USA Inc. (South Plainfield, NJ, USA). Homology searches were performed using the GenBank server of the National Center for Biotechnology Information (NCBI; http://blast.ncbi.nlm.nih.gov/Blast.cgi (accessed on 31 May 2023) and the BLAST (blastn) algorithm [38].

### 2.5. Amplicon Analysis of Bacterial 16S rRNA and Fungal ITS Genes

Samples from DNA extracted from rhizosphere soils were analyzed by next-generation sequencing of bacterial and fungal ribosomal genes. Next-generation sequencing was performed by Azenta Life Sciences (South Plainfield, NJ, USA) using an Illumina MiSeq protocol (Illumina, San Diego, CA, USA) [24]. Bacteria were identified using primers targeting the hypervariable V3 and V4 regions of the 16S rDNA gene. Fungal 18S and ITS rDNA sequences were analyzed to identify the composition and abundance of fungi. Operational taxonomic units (OTUs) were grouped based on a 97% identity threshold for data statistics and analysis. Bioinformatic analysis was performed as previously described using Qiime (1.9.1) [24]. Venn diagrams were calculated as described by Behnke-Borowczyk et al. [29]. The Jaccard similarity index was calculated as described by Real and Vargas [39].

## 3. Results

### 3.1. Tree Identification Using Barcode Genes

This study used previously reported DNA primers targeting nuclear plant ITS and chloroplast DNA barcodes for genus and species identification of trees. Identification was considered valid at the genus or species level if similar results were found with at least two barcodes. To maximize accuracy, we targeted three different genes: the internal transcribed spacer (ITS1) region of the 18S–5.8S–26S nuclear ribosomal cistron, the second subunit of the nuclear internal transcribed spacer (ITS2), and chloroplast *trnL* (UAA) intron. Standard DNA extraction and PCR procedures were modified to optimize the DNA recovery and amplification. Table 1 displays the identification of all four trees using the three target genes. We observed a 75% accuracy rate for genus and 50% for species-level identification. The three DNA barcodes did not agree on T1 classification. However, the ITS2 and UAA showed similar identification at the genus level. Both genes showed *Quercus* to be the genus for T1, but the species-level identification was not the same. ITS2 identification was *Q. planipocula*, while UAA showed *Q. rubrum*. The identification by ITS1 barcoding was *F. sylvatica*.

All barcodes used identified T3 and T4 at the genus and species level. T3 was identified as *Acer rubrum*, while T4 was identified as *F. sylvatica*. Analysis for T2 did not show any similarities between the used barcodes. The ITS1 results did not match with the ITS2 and UAA. All three barcodes showed different genera and species names.

### 3.2. α Diversity Analysis of Bacteria OTU

**Figure 1:**
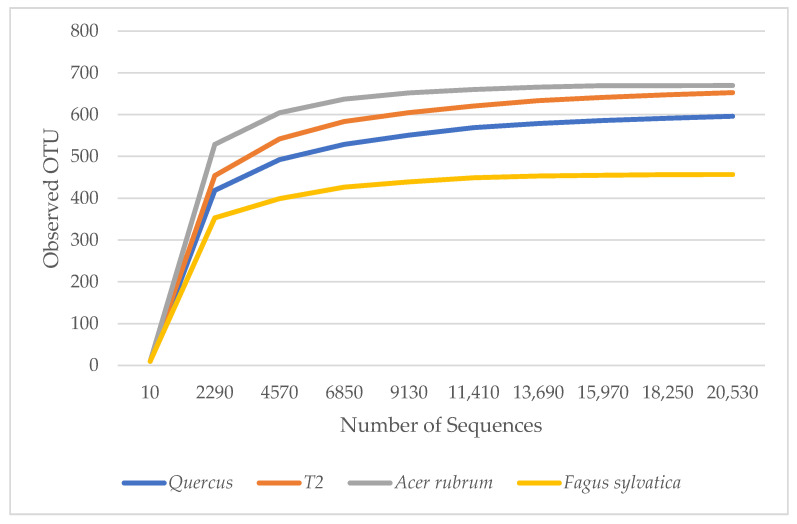
Rarefaction measure: observed bacteria OTU species over sequences per sample.

α diversity is used to reflect the diversity of each sample, which estimates the number of species in the microbial community and the abundance and diversity of species in environmental communities. The rarefaction curves in Figure 1 show the number of species in each sample. The curves demonstrated that the numbers of sequences analyzed were sufficient to predict species abundance. *A. rubrum* showed the highest diversity, followed by T2, *Quercus*, and *F. sylvatica*.

### 3.3. Rhizosphere Bacterial Communities Based on Bacterial Phyla

**Figure 2:**
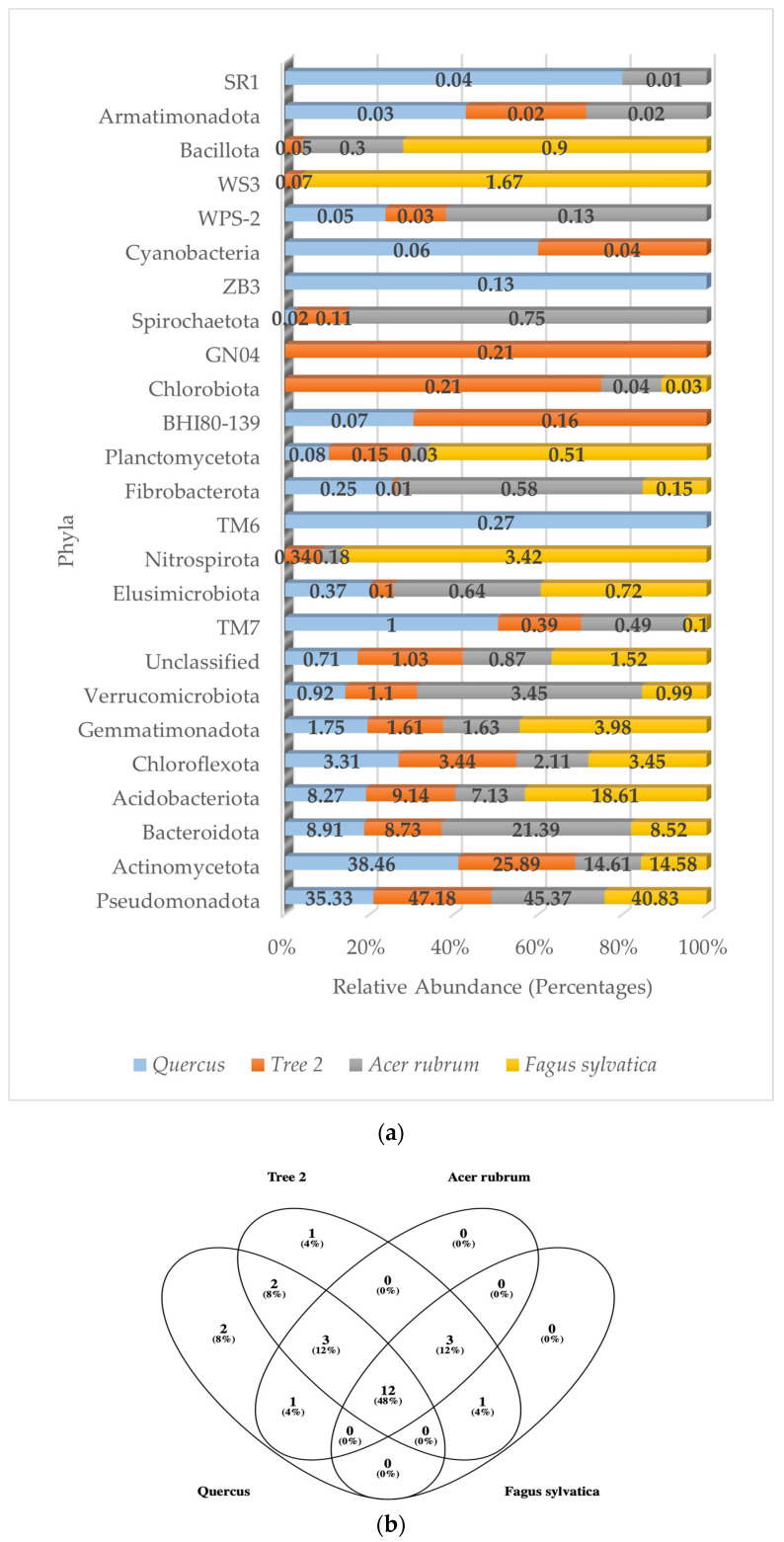
(**a**) Relative abundance of dominant bacterial phyla in the rhizosphere; (**b**) Venn diagram of common bacterial phyla.

A total of 24 bacterial phyla, 55 classes, 74 orders, 101 families, and 67 genera were detected in the rhizosphere. Of the 24 phyla detected, 16 (67%) showed relative abundance values below 1%, while the Actinomycetota, Pseudomonadota, Bacteroidota, Chloroflexota, and Acidobacteriota accounted for an average of 91% in all trees. Figure 2a shows the relative abundance of the dominant bacterial phyla corresponding to *Quercus*, T2, *A. rubrum*, and *F. sylvatica*, respectively. The numbers of bacterial phyla detected were 16 (*F. sylvatica*), 20 (*Quercus*), and 23 (T2 and *A. rubrum*).

The composition and relative abundance of the dominant bacterial phyla in *Quercus* (by descending order) was Actinomycetota (38.46%), Pseudomonadota (35.33%), Bacteroidota (8.91%), Acidobacteriota (8.27%), Chloroflexota (3.31%), and Gemmatimonadota (1.75%). All other phyla values were less than 1%.

In T2, the distribution of bacterial phyla was Pseudomonadota (47.18%), Actinomycetota (25.89%), Acidobacteriota (9.14%), Bacteroidota (8.73%), Chloroflexota (3.44%), and Gemmatimonadota (1.61%). All other phyla values were less than 1.2%.

The dominant bacterial phyla in *A. rubrum* were Pseudomonadota (47.37%), followed by Bacteroidota (21.39%), Actinomycetota (14.61%), Acidobacteriota (7.13%), Verrucomicrobiota (3.45%), and Chloroflexota (2.11%). All other values were less than 1.7%.

The *F. sylvatica* bacterial community was based on the following dominant phyla: Pseudomonadota (40.83%), Acidobacteriota (18.61%), Actinomycetota (14.58%), Bacteroidota (8.52%), Gemmatimonadota (3.95%), Chloroflexota (3.45%), and Nitrospirota (3.42%). All other phyla were less than 1.6%.

There were 12 common bacterial phyla shared by all trees (Figure 2b). They were the Pseudomonadota, Actinomycetota, Bacteroidota, Acidobacteriota, Chloroflexota, Gemmatimonadota, Verrucomicrobiota, TM7, Elusimicrobiota, Fibrobacterota, Planctomycetota, and unclassified sequences.

A similarity value of one between trees indicated that bacterial communities had the same composition at the phylum level. The closer to one, the more similar they are. The values were *Quercus*/*F. sylvatica* (0.50), *Quercus*/*A. rubrum* (0.73), *Quercus*/T2 (0.64), T2/*A. rubrum* (0.78), T2/*F. sylvatica* (0.73), and *A. rubrum*/*F. sylvatica* (0.75). The highest similarity was between T2 and *A. rubrum*. The lowest was between *Quercus* and *F. sylvatica*. The average similarity value between trees at the phylum level was 0.69.

### 3.4. Rhizosphere Bacterial Communities Based on Top 10 Bacterial Genera and Order

**Figure 3:**
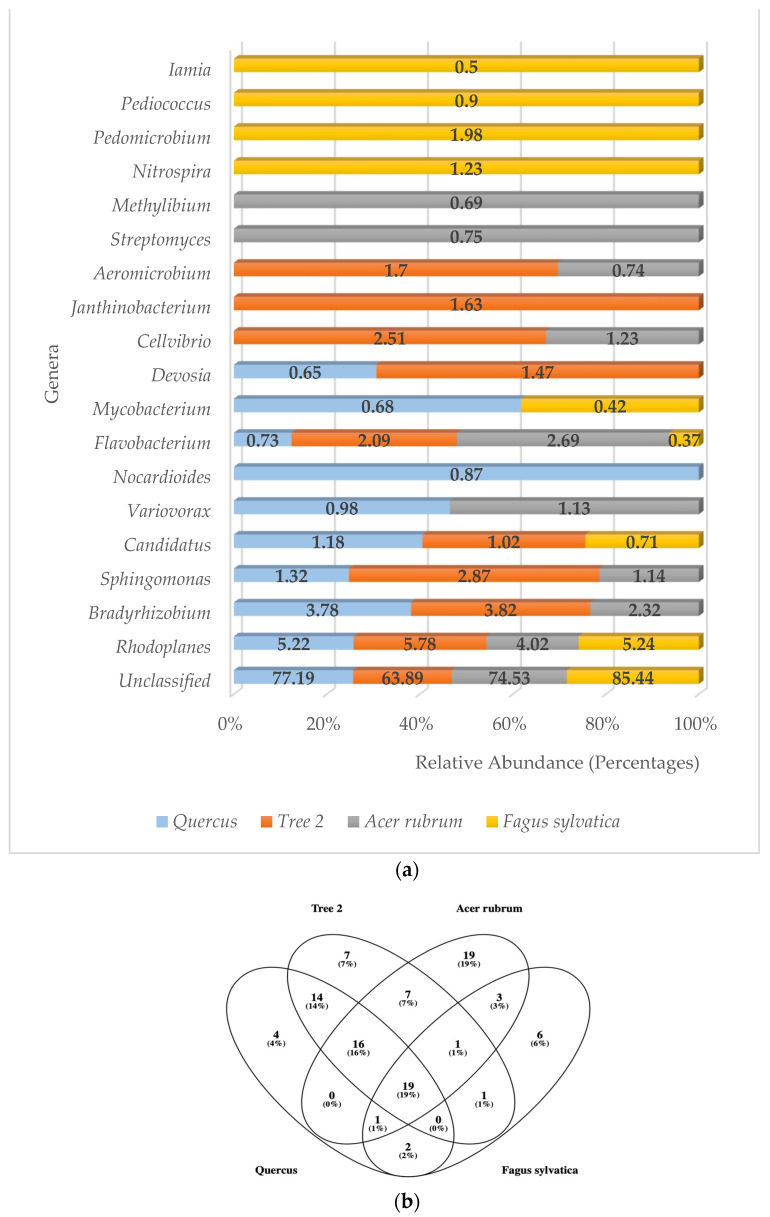
(**a**) Relative abundance of predominant bacterial genera in the rhizosphere; (**b**) Venn diagram of common bacteria genera.

A total of 67 genera were detected in the rhizosphere. *A. rubrum* showed the highest number of genera (67), followed by Tree 2 (65), *Quercus* (56), and *F. sylvatica* (33), respectively. Unclassified bacterial genera were found to be the number one type in all trees with an average of 76.26% (Figure 3a). The phylum Pseudomonadota had more genera with high levels of relative abundance. These were *Rhodoplanes*, *Bradyrhizobium*, *Sphingomonas*, *Variovorax*, *Devosia*, *Cellvibrio*, *Janthinobacterium*, *Methylibium*, *Pedomicrobium*, and *Mesorhizobium*, followed by the Actinomycetota (5) (*Iamia*, *Streptomyces*, *Aeromicrobium*, *Nocardioides*, *Mycobacterium*), Bacteroidota (1) (*Flavobacterium*), and Nitrospirota (1) (*Nitrospira*).

When identification was possible, the genus *Rhodoplanes* was the most frequently detected at higher relative abundance levels in all trees (5.22%, 5.78%, 4.02%, and 5.24%, respectively), followed by *Bradyrhizobium* (3.78%, 3.82%) in *Quercus* and T2, *Flavobacterium* (2.69%) in *A. rubrum*, and *Pedimicrobium* (1.98%) in *F. sylvatica*. The third most abundant genera were *Sphingomonas* (1.32%, 2.87%) in *Quercus* and T2, *Flavobacterium* (2.69%) in *A. rubrum*, and *Nitrospira* (1.23%) in *F. sylvatica.*

Bacteria belonging to the genera *Rhodoplanes* and *Flavobacterium* were the only ones detected with high levels of abundance in all trees. The genera *Bradyrhizobium* and *Sphingomonas* were part of the dominant community in *Quercus*, T2, and *A. rubrum* but were absent in *F. sylvatica*. Five different genera only showed high levels of abundance in *F. sylvatica*. These were *Nitrospira*, *Pedomicrobium*, *Pediococcus*, *Iamia*, and *Mesorhizobium*. The genera *Methylibium* and *Streptomyces* were only detected in *A. rubrum*. *Janthinobacterium* was only detected in T2, while *Nocardioides* was only found in *Quercus*.

When total bacterial genera were analyzed to determine the core microbiome in all trees, 19 were found to be present in all samples (Figure 3b). These genera were *Rhodoplanes*, *Sphingomonas*, *Flavobacterium*, *Cellvibrio*, *Candidatus*, *Devosia*, *Variovorax*, *Nocardioides*, *Mycobacterium*, *Iamia*, *Pedomicrobium*, *Methyllibium*, *Hypomicrobium*, *DA101*, *Phenylbacterium*, *Kaistobacter*, *Mesorhizobium*, and *Afifella.* All samples shared the unclassified category.

The Jaccard similarity index analysis for the most abundant genera showed that *F. sylvatica* was very different from the other trees. The values were *Quercus*/*F. sylvatica* (0.33), *Quercus*/*A. rubrum* (0.43), *Quercus*/T2 (0.54), Tree 2/*A. rubrum* (0.54), T2/*F. sylvatica* (0.25), and *A. rubrum*/*F. sylvatica* (0.18). The highest similarity was between T2 and *A. rubrum*. The lowest was between *A. rubrum* and *F. sylvatica*. The average similarity value between trees at the genus level was 0.38.

Because most of the genera detected were unclassified, we analyzed higher taxonomical levels to try to have a better understanding of the community structure in the rhizosphere. The levels of unclassified bacteria were reduced to an average of 10.96% at the order level. *A. rubrum* showed the highest number of orders (74), followed by *Quercus* (70), T2 (69), and *F. sylvatica* (68), respectively.

**Figure 4:**
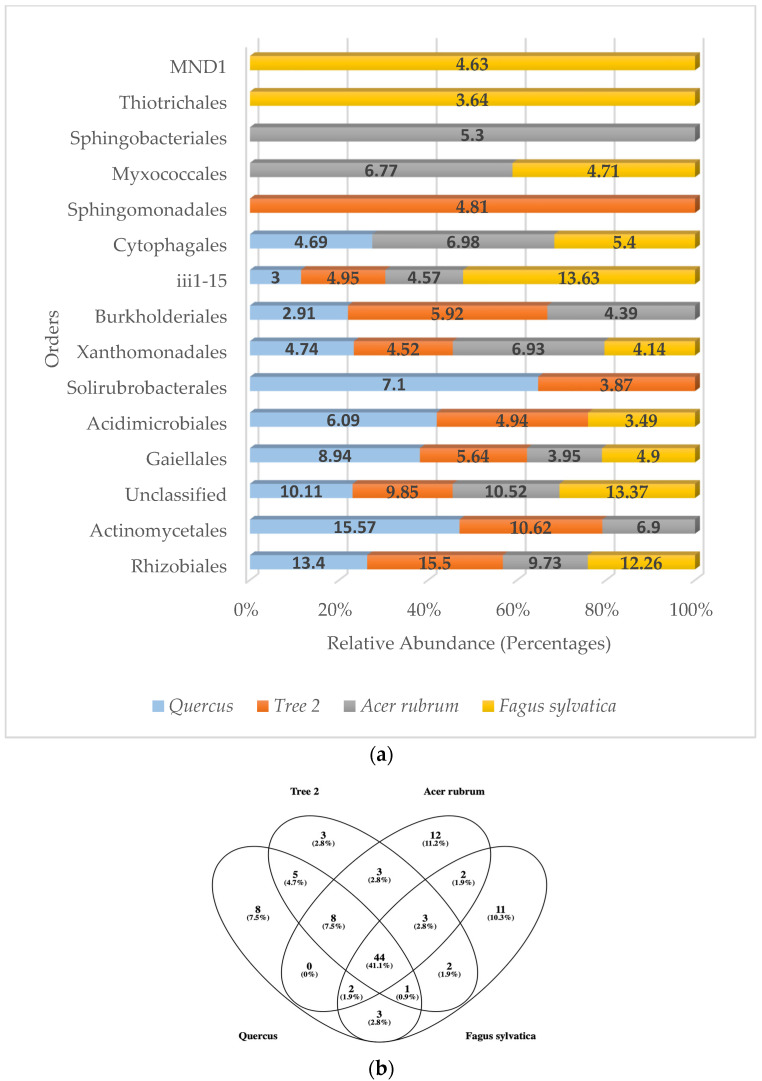
(**a**) Relative abundance (percentage) of predominant bacterial orders in the rhizosphere; (**b**) Venn diagram of common bacterial orders.

When analyzing the most frequently detected orders by categories, six belonged to the Pseudomonadota phylum (Rhizobiales, Xanthomonadales, Burkholderiales, Sphingomonadales, Thiotrichales, MND1), four to the Actinomycetota (Gaiellales, Actinomycetales, Acidimicrobiales, Solirubrobacterales), one to the Acidobacteriota (iii1-15), two to the Bacteroidota (Cytophagales, Sphingobacteriales), and one to the Myxococcota (Myxococcales) (Figure 4a).

The most abundant bacteria detected at the order level in *Quercus* was the Actinomycetales (15.57%), followed by Rhizobiales (13.40%) and unclassified sequences (10.11%). In T2, Rhizobiales (15.50%) were number one, followed by Actinomycetales (10.62%) and unclassified (9.85%). *A. rubrum* showed unclassified (10.52%) as the number one sequence detected, followed by Rhizobiales (9.73%) and Actinomycetales (6.9%). In *F. sylvatica*, iii1-15 (13.63%) was dominant, followed by unclassified sequences (13.37%) and Rhizobiales (12.26%). There were four bacterial orders that were found in the dominant community of all trees. They were the Rhizobiales, Gaiellales, Xanthomonadales, and iii1-15. When analyzing the total bacterial orders detected, the results showed that 44 orders were found in all samples (Figure 4b).

Similarity values for the most abundant orders were *Quercus*/*F. sylvatica* (0.53), *Quercus*/*A. rubrum* (0.67), *Quercus*/T2 (0.82), T2/*A. rubrum* (0.54), T2/*F. sylvatica* (0.43), and *A. rubrum*/*F. sylvatica* (0.54). When it came to the similarity of dominant orders between trees, *F. sylvatica* showed the lowest similarity with T2. The highest similarity values were found between *Quercus* and T2. The average similarity value at the order level between trees was 0.59.

### 3.5. α Diversity Analysis of Fungi OTU

**Figure 5:**
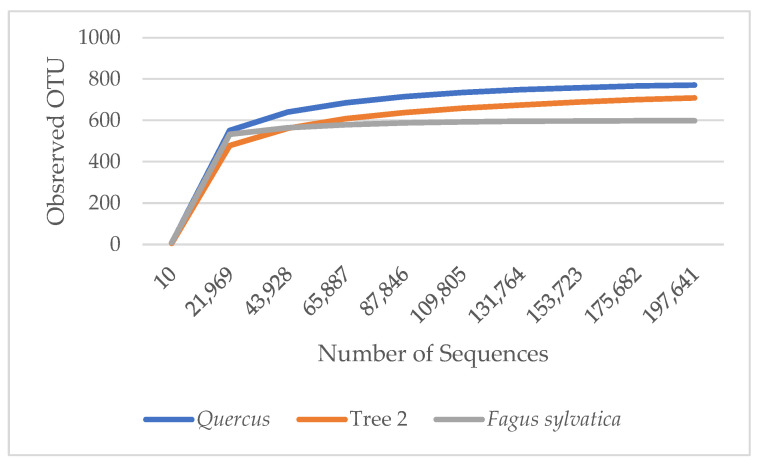
Rarefaction measure: observed fungi OTU species over sequences per sample.

The rarefaction curves in Figure 5 show the number of fungal species in each sample, demonstrating that the number of sequences analyzed was sufficient to predict species abundance. Rarefaction curves showed *Quercus* with the most diverse fungi community, followed by T2 and *F. sylvatica*. *A. rubrum* fungi characterization was not performed due to insufficient DNA concertation to complete the analysis.

### 3.6. Rhizosphere Fungal Communities Based on Phyla

A total of 10 fungal phyla, 26 classes, 59 orders, 96 families, 140 genera, and 109 species were detected in the rhizosphere. More than 83.49% of fungi detected belonged to the phyla Ascomycota and Basidiomycota.

**Figure 6:**
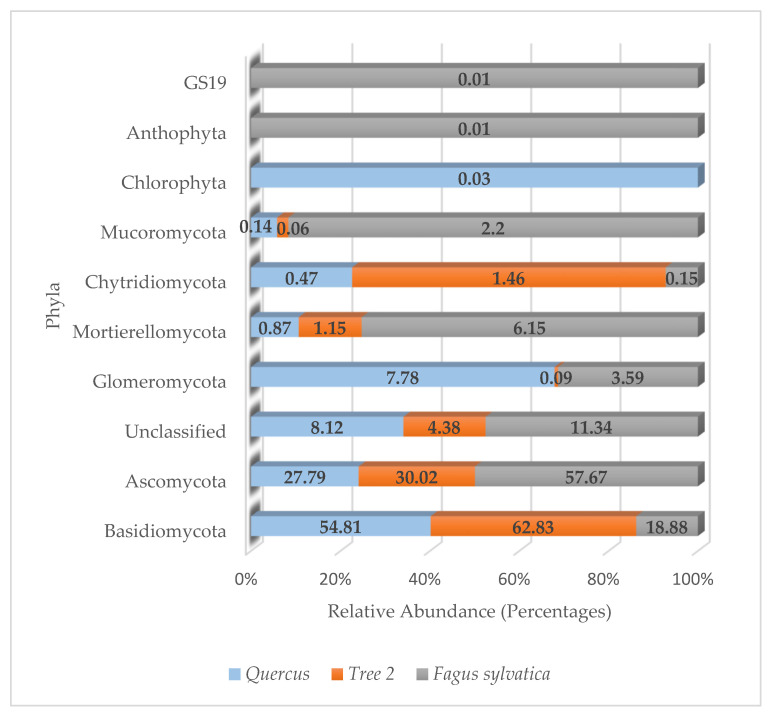
Relative abundance of fungi phyla in the rhizosphere.

The distribution of fungal phyla in trees is described in Figure 6. The relative abundance in *Quercus* was Basidiomycota (54.81%), Ascomycota (27.79%), unclassified (8.12%), and Glomeromycota (7.78%). All other fungi phyla were below 1%.

T2 showed a relative abundance dominated by Basidiomycota (62.83%), followed by Ascomycota (30.02%), unclassified sequences (4.38%), Chythridiomycota (1.46%), and Mortierellomycota (1.15%), with all others below 1%.

The fungal community in *F. sylvatica* was dominated by Ascomycota (57.67%), followed by Basidiomycota (18.88%), unclassified (11.34%), Mortierellomycota (6.15%), Glomeromycota (3.59%), and Mucoromycota (2.2%), with all others below 1%.

Seven out of the ten phyla were detected in all trees. They were unclassified sequences, Ascomycota, Basidiomycota, Glomeromycota, Mortierellomycota, Chytridiomycota, and Mucoromycota. The phyla Anthophyta and GS19 were only present in *F. sylvatica*, while Chlorophyta was only detected in *Quercus*.

Similarity values for fungi phyla were *Quercus*/T2 (0.88), *Quercus*/*F. sylvatica* (0.70), and T2/*F. sylvatica* (0.78). The lowest similarity value at the phylum level was observed between *Quercus* and *F. sylvatica*. The highest was between *Quercus* and T2. The average similarity values at the phylum level were 0.79.

### 3.7. Rhizosphere Fungal Communities Based on Genera

**Figure 7:**
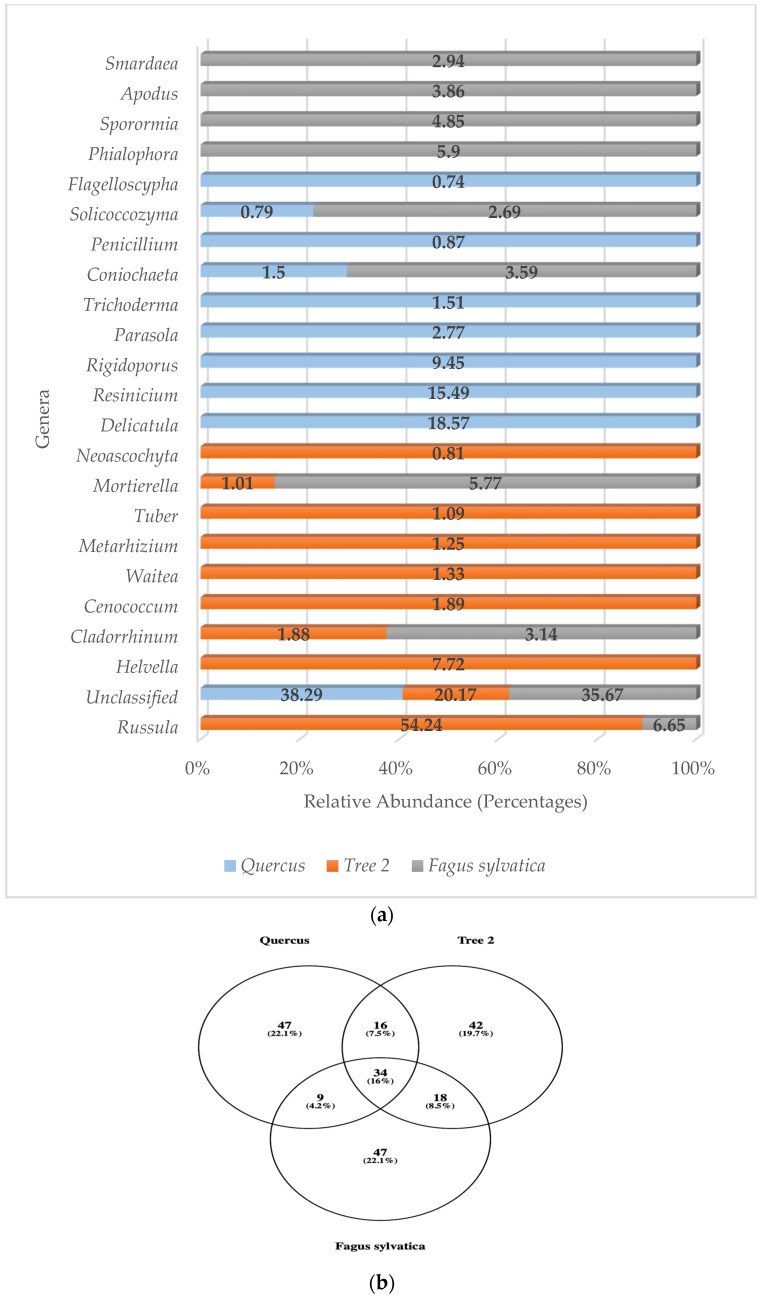
(**a**) Relative abundance of predominant fungi genera; (**b**) Venn diagram of common fungi genera.

Identification at the species level averaged 72.1% of unclassified fungal sequences, so we used genera to ascertain the fungi community structure in the rhizosphere. The number of unclassified fungi dropped to 31.38% using the genus level. A total of 140 fungal genera were detected in the rhizosphere, with T2 showing the highest numbers with 110, followed by *F. sylvatica* (108) and *Quercus* (106). The distribution for the most abundant genera is described in Figure 7a.

Most of the dominant fungi genera with high relative abundance were members of the Ascomycota. These were Coniochaeta, Sporormia, Metarhizium, Penicillium, Apodus, Helvella, Tuber, Smardaea, Cladorrhinum, Cenococcum, Neoascochyta, Trichoderma, and Phialophora. Basidiomycota fungi genera accounted for eight of the most frequently detected sequences (*Russula*, *Waitea*, *Delicatula*, *Resinicium*, *Rigidoporus*, *Solicoccozyma*, *Parasola*, *Flagelloscypha*). One more genus, *Mortierella*, was detected with high relative abundance, but it belonged to the phylum Mortierellomycota.

Unclassified sequences were predominant in *Quercus* and *F. sylvatica*, while *Russula* was the most abundant mold in T2, contributing 54.24% of the community. In T2, *Russula* was followed by *Helvella* (7.72%) and *Cenococcum* (1.89%). *Phialophora* (5.90%) and *Mortierella* (5.77%) completed the top three genera in *F. sylvatica*. The number one genus detected in *Quercus* was *Delicatula* (18.57%), followed by *Resinicium* (15.49%) and *Rigidoporus* (9.45%).

No mold was detected in the top 10 sequences of all trees. Of the most abundant genera, only *Russula*, *Cladorrhinum*, *Mortierella*, *Coniochaeta*, and *Solicoccozyma* were detected in at least two samples (T2 and *F. sylvatica*). Looking at the total genera, 34 were detected in all trees (Figure 7b).

Similarity values for the most abundant fungi genera were *Quercus*/T2 (0.05), *Quercus/F. sylvatica* (0.18), and T2/*F. sylvatica* (0.25). Similarity analysis showed that the lowest similarity was between *Quercus* and T2, and the highest was with T2 and *F. sylvatica* (0.25). The average similarity value was 0.16.

## 4. Discussion

Eukaryotic organisms are complex; genetic inheritance patterns can be misleading. While the ITS2 is a subunit of the ITS1, they display different phylogenies [9,37]. They are different genes and, therefore, subjected to different evolutionary pressures. We cannot rely on one genetic sequence to be used as a barcode to identify land plants and trees. In this study, we used two nuclear genes and one chloroplast gene amplified by PCR with previously reported primers to identify four different trees at BCC [7,9,36]. The ITS is preferred for its universality at the sacrifice of specificity [2,7,36]. Human error and other confounding variables must be accounted for when considering the significance of this study. DNA extractions rely on a successful procedure, which we modified to accommodate the complexity of plants. Previous studies using chloroplasts, DNA barcodes, *rbcLa*, and *trnH-psbA* to identify African trees demonstrated that the combination of multiple barcodes provided a more accurate identification at the genus and species level when compared to morphological methods [40,41]. However, some of the samples were not identified at the species level due to a lack of genetic information in the databases and a lack of discriminatory power of the barcodes used to differentiate closely related genera and species. When a low species-to-genus ratio was found in trees, DNA barcode identification was significantly enhanced for African tree species in montane forests [40]. The identification of trees was higher at the genus level than at the species level. In our study, the ITS2 and UUA barcodes identified the genus *Quercus*, but species identification was different. The ITS1 did not match either genus or species. T2 identification did not show any match between barcodes at the genus or species level. Nevertheless, in our study, we were able to have all three barcodes identifying *A. rubrum* and *F. sylvatica*, respectively.

*A. rubrum* belongs to the family Aceraceae, which has the largest number of broad-leaved deciduous trees in the Northern Hemisphere. Maple species are widely distributed in forest ecosystems [1,42]. Efficient DNA barcode identification of Aceraceae trees demonstrated the use of the ITS and chloroplast sequences [42]. It reported that the nuclear ITS genes were more accurate than the maternally inherited chloroplast barcodes. Because of the complexity of sexual reproduction in Aceraceae due to interspecific hybridization and introgression, barcoding can be very challenging. However, in our study, the two ITS and the chloroplast UAA barcodes could identify *A. rubrum* accurately at the genus and species levels.

*F. sylvatica* (European beech) is a widely distributed temperate tree species across Europe due to its adaptation to different types of soils and weather. However, it has a very high sensitivity to high temperatures and drought [25]. It also has a high tolerance to pH and water content compared to other trees. The molecular identification of *F. sylvatica* was reported with the same UUA barcodes used in this study and polymerase chain reaction-restriction fragment length polymorphism (PCR-RFLP) using DNA extracted from fine roots [43]. Other studies demonstrated the use of mitochondrial genome sequences to barcode different species, such as *F. sylvatica*, *F. crenata*, and *F. engieriana* [44].

*Quercus* is a North American tree species [18]. In the Northern Hemisphere, temperate forests are inhabited by trees of the Fagaceae and Pinaceae. In this study, we identified *Quercus* at the genus level, but species discrimination was variable. Plant barcoding of *Quercus* species can be very challenging because gene exchange can happen due to hybridization and polyploidy, complicating the differentiation between species. It can also be affected by the low molecular evolution because of the absence of polymorphisms between genera. Many oak species, such as *Quercus*, can hybridize, and their chloroplast halotypes are shared between species [45,46]. *Quercus* taxonomy is further complicated by the homologous interspecies variations due to reproductive isolation and ecological adaptations to different environmental fluctuations. Therefore, chloroplast sequences might not be very reliable in some situations requiring the use of nuclear genes, such as the ITS, to separate different species within the same genus [5,47]. When chloroplast sequences were not capable of accurately identifying of *Quercus* species in Italy, ITS sequences provided greater resolution and specificity to distinguish closely related species [47]. Recent advances in next-generation sequencing allowed the use of the whole chloroplast genome, resulting in accurate species identification [46]. Other studies demonstrated the use of nuclear DNA markers to identify and discriminate between *Q. robur* and *Q. petrea*, which were very difficult to differentiate by morphological and phenotypic analyses [48]. A combination of four primer pairs targeting nuclear sequences was good enough to enhance the discriminatory power of the assay. We plan to use additional plastid and ITS barcodes to optimize tree identification at the study site. Further analysis will also be performed with additional trees at different locations to identify their genotype.

Amplicon analysis of the rhizosphere microbiome using ribosomal genes was performed to ascertain the community structure, diversity, and abundance of bacteria and fungi in all four trees. Similarity values demonstrated that the bacterial communities from each tree became more different when lower taxonomical levels, such as orders and genera, were analyzed. At the phylum level, the Proteobacteria dominated the rhizosphere bacterial communities of *F. sylvatica*, *A. rubrum*, and T2. The rhizosphere of *Quercus* was the only one with Actinomycetota as the main phylum, followed by Pseudomonadota. Pseudomonadota bacteria orders and genera were more dominant in all trees. These bacteria are known to be capable of diverse metabolic activities, such as nitrogen fixation, organic matter decomposition, nutrient mobilization, plant growth promotion, etc. [1,11,14,23]. Actinomycetota orders and genera were also widely distributed and were commonly associated with organic matter decomposition, secondary metabolites production, organic matter decomposition, plant growth promotion, etc. Both bacterial phyla were previously shown to be major contributors in the rhizosphere of *Quercus* and *F. sylvatica* [1,25].

High variability was found between trees based on similarity values. For instance, in *Quercus*, the second most abundant bacterial phylum was Pseudomonadota, and in *F. sylvatica*, it was Acidobacteriota. *A. rubrum* bacteria associated with the Bacteroidota were enriched in the rhizosphere. Similar results were reported in different studies, where relative abundance values fluctuated between different trees and locations [20,49,50,51,52]. Previous studies showed that the community structure of the rhizosphere of six plant species was also dominated by bacteria belonging to the Pseudomonadota, Actinomycetota, Bacteroidota, and Acidobacteriota [20]. In this study, a core microbiome at the phylum level consisted of Pseudomonadota, Actinomycetota, Bacteroidota, Acidobacteriota, Chloroflexota, Gemmatimonadota, Verrucomicrobiota, TM7, Elusimicrobiota, Fibrobacterota, and Planctomycetota. These phyla have metabolically diverse bacteria that perform the same chemical reactions in the rhizosphere and are functionally redundant. All of them have bacteria that decompose organic compounds, fix carbon, fix nitrogen, weather minerals, etc. All these biochemical reactions promote tree health and stability [11,14,23]. In *Q. pyrenaica*, the bacterial community structure was dominated by Pseudomonadota, Acidobacteriota, Bacteroidota, and Verrucomicrobiota [53]. In *F. sylvatica*, the rhizosphere core microbiome was reported to be based on Acidobacteriota and Actinomycetota, the class Alphaproteobacteria, and the genera *Mycobacterium*, *Bradyrhizobium*, and *Rhodoplanes* [25].

Previous studies in soils at BCC demonstrated that the average relative abundance for bacterial phyla in surface soils was Actinomycetota (33.76%), Pseudomonadota (25.59%), Chloroflexota (9.67%), Acidobacteriota (8.98%), Planctomycetota (4.87), Bacteroidota (4.14%), Verrucomicrobiota (3.56%), Cyanobacteria (2.46%), and Gemmatimonadota (2.29%). All other phyla accounted for less than 2% [24]. When compared to the average numbers found in bulk soil at BCC, the average relative abundance of Pseudomonadota in the rhizosphere increased to 42.18%. No other bacterial phyla showed such a dramatic increase. Bacteroidota increased to 11.89% and Acidobacteriodota to 10.79%. On the other hand, Actinomycetota and Chloroflexota decreased to 23.39% and 3.08%, respectively. All other phyla showed levels below 2.4%. Similar results were reported with different phyla enriched in the rhizosphere when compared to bulk soils, where the acidification of the rhizosphere by plants and trees selected for specific taxa reduced the bacterial diversity but increased microbial biomass [1,11,20,23,25,50,51,52].

Root exudation by trees is based on the release of organic acids, carbohydrates, and amino acids, which will select and promote the growth of specific microorganisms to improve nutrient acquisition, nutrient cycling, and plant health [1,23]. The great majority of plants decrease soil pH, which results in nutrient mobilization, but *F. sylvatica* can lower it to levels that might affect the bacterial diversity. This is because its high tolerance to environmental fluctuations, such as pH, nutrient availability, and water content, requires the selection of specific bacteria to enhance nutrient access [25]. In the current study, *F. sylvatica* showed the highest abundance of Acidobacteriota but it also showed the less diverse bacterial community and the highest numbers of unclassified bacterial phyla, orders, and genera, while Colin et al. [25] showed Acidobacteriota numbers decreasing in rhizosphere samples when compared to bulk soils. Bacteria belonging to the Acidobacteriota thrive in nutrient-poor soils where they can improve plant health by mobilizing organic materials and minerals usually unavailable for plant growth [25]. Acidobacteria have been previously shown to be oligotrophic bacteria adapted to slow growth in acidic environments in the rhizosphere of *F. sylvatica* [19,25,26,51,54]. In this study, *F. sylvatica* also showed lower levels of similarity with all other trees with different bacterial composition and abundance values at the genus and order levels.

On the other hand, *A. rubrum* showed the most diverse bacterial community of all trees with the highest abundance of Bacteroidota orders and genera. Bacteria belonging to the Bacteroidota are copiotrophic organisms capable of fast growth where resources are abundant [1,11,23,50]. They can also decompose complex polymers, promote plant growth, and mobilize phosphorus (P) in soils. High diversity in the rhizosphere is correlated with root exudate and litter decomposition [1,55]. A high concentration of organic matter will select copiotrophic bacteria in the rhizosphere. *A. rubrum* was the only tree that enriched high numbers (double digits) of Bacteroidota when compared to *Quercus*, *F. sylvatica*, and T2. It was reported that maple trees, such as *A. rubrum*, did not affect soil acidification as much as *F. sylvatica* and *Quercus* [1,24]. This might have affected the diversity and composition of *Quercus* and *F. sylvatica*, showing less diverse rhizosphere bacterial communities compared to *A. rubrum* and T2 because pH is the most important factor controlling bacterial diversity in soils [1,13,22,23,27]. In this study, we did not measure soil pH or perform any soil chemistry analysis. Future studies will analyze the soil chemistry and pH to determine the chemical composition of the rhizosphere soil for each tree. To the best of our knowledge, this is the first study describing the rhizosphere bacterial community of *A. rubrum* using an analysis of 16S rRNA genes.

Unclassified sequences showed the highest relative abundance at the genus level in all trees, with more than 75% not matching any known bacteria. This demonstrated the inability of the current databases to identify bacteria in bulk soil and rhizospheres [1,24,25,27,51,52]. Most bacteria in the soil and rhizosphere have not been cultivated. Furthermore, no reference sequences are available at different taxonomical levels. However, when identification was possible at the genus and order level, the core microbial genome in the rhizosphere of all trees at the genus level consisted of 19 different genera and 45 orders. Previous studies showed that in cotton weed trees, the core microbiome consisted of 35 OTUs, with bacteria belonging to the orders Rhizobiales and Burkholderiales [49]. In our study, most bacteria were associated with the orders Rhizobiales, Cytophagales, Actinomycetales, Myxococalles, and Xanthomonadales. Relative abundance values were different between all trees. For instance, bacteria belonging to the order Rhizobiales were dominant in T2 and *A. rubrum*. However, in *Quercus*, Actinomycetales became the most abundant bacterial order. Order iii1-15, which belongs to the Acidobacteriota phylum, was enriched in *F. sylvatica*. It is usually associated with low pH and organic carbon concentration soils [54]. Genomic analysis of Acidobacteriota isolates demonstrated the presence of single or double copies of 16S rRNA genes, which may indicate slower growth rates typical of oligotrophic bacteria.

When identification was possible at the genus level, the highest percentage of relative abundance in all trees was associated with the genus *Rhodoplanes*. *Rhodoplanes* were previously reported to be important members of the rhizosphere in plants and trees [15,25,56,57,58,59,60]. *Rhodoplanes* belong to the phylum Pseudomonadota of the order Rhizobiales within the class Alphaproteobacteria. They are purple non-sulfur phototrophic and chemo-organoheterotrophic bacteria. Growth is possible both chemotropically under aerobic conditions in the dark or under anaerobic conditions by denitrification and photoheterotrophically. Previous studies suggested the possibility of nitrogen fixation capabilities in the genus *Rhodoplanes* [15]. The other bacteria detected in all trees at high frequencies were the genus *Flavobacterium*. The highest abundance was detected in *A. rubrum*. *Flavobacterium* is a genus of the phylum Bacteroidota associated with organic matter decomposition, plant growth promotion, phosphorus mobilization, and antimicrobial activity against plant pathogens [15]. They are classified as copiotrophic bacteria, which are fast growers and capable of degrading highly complex organic material such as cellulose, chitin, and polyphenols [1,23,61]. Copiotrophic bacteria, such as *Flavobacterium*, are adapted to nutrient-rich environments, like the rhizosphere. Plant-associated *Flavobacterium* in the rhizosphere showed a novel molecular mechanism of P turnover, which optimized the conversion of organic P (Po) to bioavailable inorganic P (Pi). This solubilizes P before it becomes available to plants and trees [61].

Two other genera belonging to the phylum Pseudomonadota were detected in high abundance in *Quercus*, T2, and *A. rubrum* but not in *F. sylvatica*. They were *Bradyrizhobium* and *Sphingomonas.* However, *Bradyrhizobium* was previously reported to be enriched in the rhizosphere of *F. sylvatica* grown in French soils, but only under very acidic conditions [25,51]. In this study, *Bradyrhizobium* was not detected at all in *F. sylvatica*, and *Sphingomonas* abundance was extremely low. *Bradyrhizobium* is a nitrogen fixer with the ability to consume complex molecules and root exudates. In addition to nitrogen, it can also fix carbon [62]. It was reported to be a dominant member in the rhizosphere of poplar trees, *Q. pyrenaica*, turfgrass lawns, and sugarcane [56,59,63,64,65]. However, in a study of the effect of urbanization on soil microbial communities, *Bradyrhizobium* was shown to be significantly reduced in *F. grandifolia* soils [66]. As urbanization increased, soil pH decreased. In that study, pH and ammonia concentration in soils were negatively correlated with the abundance of *Bradyrhizobium.* The addition of ammonia to soils inhibited nitrogenase activity, reducing the role of nitrogen-fixing bacteria in the bacterial community. *Sphingomonas* was a dominant genus in the rhizosphere of different plant species [1,20,65]. *Sphingomonas* promotes plant growth by producing different hormones and accelerating the growth of roots and root hairs [67].

The genus *Nitrospira* was only detected at high levels of abundance in *F. sylvatica*. *Nitrospira* was previously reported to be a member of the rhizosphere in *F. sylvatica* [25]. They are bacteria that are enriched in acidic soils with low pH [19]. In this study, the levels of the Nitrospirota phylum and the genus *Nitrospira* in *F. sylvatica* were three times more than in the other trees, indicating again the possible oligotrophic conditions in the rhizosphere, which may explain the low bacterial diversity compared to the other trees. *Nitrospira* can perform the complete oxidation of ammonia to nitrate, enhancing the removal of nitrogen from the rhizosphere [68,69]. Soils beneath *F. sylvatica* were reported to be more acidic due to the release of high concentrations of organic acids through root exudates [70]. Ling et al. [11] stated that genes involved in organic carbon decomposition, nitrogen fixation, and denitrification are enriched in the rhizosphere. However, nitrification genes are not enriched at the same level and, in some situations, they are depleted when organic carbon concentration increases.

When it came to fungi communities, the Ascomycota showed the highest abundance values in Tree 2 and *Quercus*. However, Basidiomycota was the most abundant phylum in *F. sylvatica*. Similar results were previously reported for *F. sylvatica*, with the Basidiomycota accounting for 81% of the fungi community in French soils, followed by the Ascomycota with 8.1% [51]. *F. sylvatica* showed the lowest fungal diversity, with *Quercus* showing the highest. Fungi communities were different between trees based on similarity values. Predominant fungal genera detected in the rhizosphere were largely saprophytic Ascomycota genera, such as *Coniochaeta*, *Sporormia*, *Metarhizium*, *Penicillium*, *Apodus*, *Helvella*, *Smardaea*, *Neoascochyta*, *Trichoderma*, and *Phialophora*, and ectomycorrhizal genera, such as *Tuber*, *Cladorrhinum*, and *Cenococcum*. Basidiomycota were also found in high abundance with saprophytic (*Waitea*, *Delicatula*, *Resinicium*, *Rigidoporus*, *Solicoccozyma*, *Parasola*, *Flagelloscypha*) and ectomycorrhizal genera (*Russula*). The only other genus detected in high numbers that did not belong to the phyla cited above was *Mortierella*, which belongs to the phylum Mortierellomycota. *Russula* was found to be the dominant genus detected in T2 and *F. sylvatica*, while *Delicatula* showed the highest abundance in *Quercus*. *Russula* and *Cenococcum*, both EC fungi, were shown to be very important members of fungi communities in the rhizosphere of *F. sylvatica* and *Quercus* [19,24,28,30,51,71,72,73]. They form symbiotic associations with trees when nutrient levels in the soil are very low. For instance, plants with EC fungi accumulated more phosphorus compounds due to the extension of the fungal mycelial network [26]. T2 was the only tree with 40% of the dominant fungi to be EC, e.g., *Russula*, *Cladorrhinum*, *Cenococcum*, and *Tuber*. More than half of the total genera detected in T2 belonged to the genus *Russula*. No other tree showed such dominance by a single fungal genus. Similar results were reported in the rhizosphere of *Geodorum*, Shepherd’s crooks, where *Russula* was the primary fungus, with a 29% relative abundance [73]. *Russula* has been reported to be enriched in soils with high concentrations of organic matter and acid or neutral pH.

Two fungal pathogens were detected at high levels in *Quercus*. They were *Resinicium* and *Rigidoporus*, while only one was detected in T2, e.g., *Waitea*. No fungal pathogens were detected in *F. sylvatica*. *Resinicium* and *Rigidoporus* are plant pathogens associated with wood rot [74]. *Waitea* is the cause of turfgrass disease, resulting in brown or yellow ring patches [75]. As previously reported, saprophytic fungi were more numerous than EC fungi in the rhizosphere of all trees [26,29]. Saprophytic fungi rely on the degradation of organic compounds in the rhizosphere to obtain carbon, while EC fungi rely on the plant host photosynthetic products. They are extremely important for the decomposition of organic matter and carbon cycling in the rhizosphere. It has been demonstrated that community diversity and composition of fungi in the rhizosphere are related to environmental factors such as pH, the carbon/nitrogen ratio, soil type, and moisture content [1,73]. Future studies will determine the chemical composition, pH, and the carbon/nitrogen ratio of rhizosphere soil to ascertain their effect on fungi communities.

Although the methods used in this study for rhizosphere microbiome analysis were highly reproducible, possible bias based on nucleic acid extraction from samples and PCR amplification must be acknowledged [31,32]. High numbers of DNA sequences retrieved from rhizosphere soils were not identified at the genus level for bacteria and fungi, indicating the inability of current databases to describe the natural microbial communities in environmental samples.

## 5. Conclusions

In conclusion, DNA barcoding of four trees at BCC identified *A. rubrum*, *F. sylvatica*, and *Quercus*. Our study demonstrated the great potential of DNA barcodes to identify the trees at BCC. However, it also showed its limitations by its inability to determine the species of *Quercus* and the genus and species of one tree. Additional barcodes will be tested to optimize genotypic identification. We found that each tree selected for specific bacterial and fungal communities in the rhizosphere with higher levels of bacterial abundance for Pseudomonadota, Actinomycetota, Bacteroidota, and Acidobacteriota. Fungi communities were mostly dominated by Ascomycota and Basidiomycota. At lower taxonomical levels, bacteria and fungi communities became more distinctive for each tree, indicating a stronger selection for specific types of microorganisms to inhabit the rhizosphere. The genus *Rhodoplanes* showed the highest relative bacterial abundance in all trees, while *Russula* was the most dominant fungal genus. Future studies will study the edaphic soil factors’ effect on bacterial and fungal diversity in the rhizosphere of different trees at BCC. Our goal is to genetically identify all trees on campus and to ascertain their rhizosphere microbiome to understand the relationship between host and microorganisms and the effect of soil edaphic factors on the establishment and maintenance of the interaction between trees and microbes.

## Figures and Tables

**Table 1:** Genetic identification of trees based on a BLAST analysis of target genes.

Tree	ITS1	ITS2	UAA
1	*Fagus sylvatica*	*Quercus planipocula*	*Quercus rubra*
2	*Acer rubrum*	*Fagus crenata*	*Zelkova serrata*
3	*Acer rubrum*	*Acer rubrum*	*Acer rubrum*
4	*Fagus sylvatica*	*Fagus sylvatica*	*Fagus sylvatica*

## Data Availability

The data presented in this study are available upon request to the corresponding author.
